# Fatigue survival and damage modes of lithium disilicate and resin nanoceramic crowns

**DOI:** 10.1590/1678-7757-2018-0297

**Published:** 2019-05-30

**Authors:** Fernanda Ferruzzi, Brunna M. Ferrairo, Fernanda F. Piras, Ana Flávia Sanches Borges, José Henrique Rubo

**Affiliations:** 1 Centro Universitário Ingá, Maringá, Paraná, Brasil.; 2 Universidade de São Paulo, Faculdade de Odontologia de Bauru, Departamento de Prótese, Bauru, São Paulo, Brasil.; 3 Universidade de São Paulo, Faculdade de Odontologia de Bauru, Departamento de Dentística, Endodontia e Materiais Dentários, Bauru, São Paulo, Brasil.

**Keywords:** Ceramics, Dental crowns, Fatigue, Computer-aided design, Polymers

## Abstract

Polymer-based composite materials have been proposed as an alternative for single unit restorations, due to their resilient and shock absorbing behavior, in contrast to the brittleness of ceramic materials that could result in failure by fracture.

**Objective:**

To evaluate the fatigue strength and damage modes of monolithic posterior resin nanoceramic and lithium disilicate glass ceramic crowns.

**Methodology:**

Twenty-six resin nanoceramic (RNC) and lithium disilicate glass ceramic (LD) 2 mm monolithic crowns (n=13) were cemented on composite resin replicas of a prepared tooth and subjected to cyclic load with lithium disilicate indenters for 2 million cycles. Specimens and indenters were inspected every 500,000 cycles and suspended when presenting fractures or debonding. Surviving specimens were embedded in epoxy resin, polished and subsurface damage was analyzed. Specimens presenting fractures or severe subsurface damage were considered as failures. Survival data was subjected to Fisher's exact test; damage modes were subjected to Mann-Whitney test (p<0.05).

**Results:**

There were no debonding, cohesive or catastrophic failures. Considering subsurface damage, 53.8% of RNC and 46.2% of LD crowns survived the fatigue test, presenting no statistical difference. Chief damage modes were radial cracks for RNC and inner cone cracks for LD, presenting no statistical difference.

**Conclusions:**

The results suggest that if debonding issues can be resolved, resin nanoceramic figures can be an alternative to posterior crowns. Although distinct, damage modes revealed potential to cause bulk fracture in both glass ceramic and resin nanoceramic crowns.

## Introduction

Full crowns have been widely used to restore extensively damaged teeth. The classic crown consists of a bilayer restoration: a strong and stiff ceramic core veneered with aesthetic porcelain. The structural reliability of this combination of materials is primarily controlled by the properties of the core,^1^ which provides stress-shielding of the veneer layer as well as of the underlying soft dentin support.^2^ However, the main complications reported for bilayer restorations are chipping of the weak ceramic veneer.^3^

Lithium disilicate (LD) glass ceramics present high flexural and fatigue strength, and fracture toughness^4^ ^–^ ^7^ when compared to other glass ceramics. These important mechanical properties associated to excellent optical properties^8^ resulting in a highly versatile material for the fabrication of both posterior and anterior restorations. Promising clinical performance is reported for LD crowns, with a 5-year survival rate comparable to metal ceramic crowns and less biological complications.^3^

For the posterior area, monolithic crowns have been proposed, since lithium disilicate optical properties exempt veneering ceramics in most cases.^9^ In this approach, marginal and internal fit, occlusal and proximal contacts may be checked in a single visit, once core and veneer are merged into a monolithic restoration. Additionally, by eliminating the veneering ceramics, these crowns seem to exhibit higher fatigue strength,^10^ delivering aesthetics and strength in a practical way.

Polymer-based composite materials have been proposed as an alternative for single unit restorations due to their resilient and shock absorbing behavior,^11^ in contrast to the brittleness of ceramic materials that could result in failure by fracture. A composite resin block for CAD/CAM (Lava Ultimate, 3M ESPE; St. Paul, MN, USA) was designed for fabrication of full and partial crowns, as well as veneers in a single visit. As a resin composite, firing processes are not required and polishing is performed using abrasive disks. It can be easily stained and repaired, if necessary, by direct composites. Lava Ultimate consists of around 80% nanoceramic fillers, specifically 20 nm silica particles, 4 to 11 nm zirconia particles and silica-zirconia nanoclusters, all embedded into a highly cross-linked polymeric matrix. Industrial manufacturing and additional curing of composites reduce the porosity and the amount of flaws, which seems to result in higher fatigue and flexural resistance in comparison to direct composites with conventional layering and curing processes.^12^ This material presented high fatigue strength when compared to glass-ceramics, and apparently meets the mechanical requirements for high stress-bearing areas.^6^ ^,^ ^13^ ^,^ ^14^ Despite these promising results, debonding cases were reported for composite crowns cemented on zirconia abutments^15^ ^,^ ^16^ and the manufacturer opted to change the indications, limiting the material to partial crowns and veneers. Although bond strength studies do not report problems on adhesion to RNC compared to ceramics,^17^ ^,^ ^18^ if debonding issues can be resolved, RNC figures as an esthetic, fast, repairable and resistant alternative to posterior crowns. The mechanical properties of RNC were not fully addressed; no clinical performance of tooth supported restorations was investigated.

Although clinical trials are the most reliable way to assess if mechanical properties of biomaterials will be in fact translated into clinical longevity, well-designed laboratory tests can help to predict the behavior of dental restorations, since they emulate as closely as possible the conditions encountered in the oral environment.^1^ ^,^ ^19^ Molar crowns are subjected to a fatigue process during masticatory function, with high loads and in a wet environment. This fatigue process can lead to fractures or debonding, typically considered the “worst case scenario”. Thus, the purpose of this study was to investigate the fatigue survival of monolithic posterior resin nanoceramic and lithium disilicate glass ceramic crowns and the damage modes produced by the fatigue test. The null hypothesis is that there is no influence of restorative material in the fatigue strength and damage modes of resin nanoceramic and lithium disilicate monolithic posterior crowns.

## Methodology

### Specimen's preparation

A mandibular left first molar was anatomically reduced by 1.5 mm in axial surfaces and 2 mm in occlusal surfaces for full crown preparation. Impressions of the prepared, adjacent, and opposing teeth were made (Express, 3M ESPE; St Paul, MN, USA); casts were articulated and scanned (InEos Blue, Sirona Dental Systems; Long Island City, NY, USA). Monolithic CAD/CAM lithium disilicate (n=13) (e.max CAD, Ivoclar Vivadent;Liechtentein, Germany) and resin nanoceramic crowns (n=13) (Lava Ultimate Restorative, 3M ESPE; St Paul, MN, USA) with identical anatomic contours were designed and milled in Cerec system (InLab 4.0 and MC XL, Sirona Dental Systems; Long Island City, NY, USA) with minimum occlusal thickness of 2 mm. Lithium disilicate crowns were crystallized and glazed and resin nanoceramic crowns were polished, according to manufacturer's instructions.

Resin composite dies (Z100, 3M ESPE; St Paul, MN, USA), replicas of the prepared tooth, were embedded in an acrylic resin base and stored in distilled water at 37°C for 30 days to prevent stresses from water sorption. For the luting procedure, internal surface of lithium disilicate crowns were etched with 5% hydrofluoric acid for 20 seconds. Resin nanoceramic crowns were sandblasted with 30 μm aluminum oxide at two bars for 10 seconds. All crowns were cemented to aged composite dies with an adhesive resin cement RelyX Ultimate (3M ESPE; St Paul, MN, USA) and the self-etch adhesive with silane and primers (Scothbond Universal, 3M ESPE; St Paul, MN, USA), following the manufacturer's instructions. The specimens were stored in distilled water at 37°C for a minimum of 7 days prior to mechanical testing to allow hydration of resin cement.

### Mechanical testing

**Figure 1:**
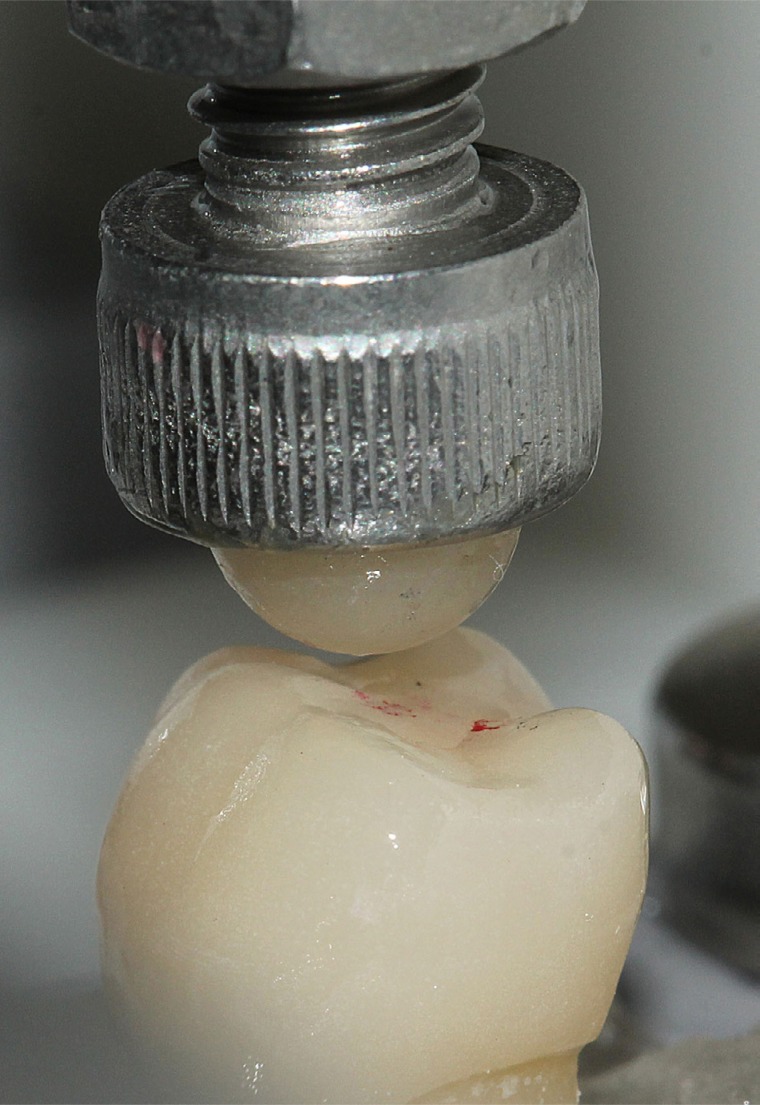
Position of indenter during fatigue test

Specimens were subjected to a mechanical fatigue test in a thermomechanical fatigue cycler (Biocycle, Biopdi; São Carlos, SP, Brazil), submersed in water at 37°C with a cyclical load varying from 0 to 350 N. Lithium disilicate (e.max Press, Ivoclar Vivadent; Liechtenstein, Germany) spherical indenters of 3,18 mm radius were manufactured by lost-wax technique and glazed according to the manufacturer's instructions. The indenters loaded the crowns at the center of the occlusal surface, between lingual and buccal cusp inclines, contacting the specimens' surface during the entire test, with no impact (Figure 1). The test was carried out at a frequency of 2 Hz, during 2 million cycles or until failure. Crowns and indenters were inspected under a stereomicroscope (MZ6 Leica; Wetzlar, Germany) with a source of light after 500,000, 1 million, 1.5 million and 2 million cycles. Specimens presenting debonding, catastrophic fracture (bulk fracture) or cohesive fracture (chipping) were considered as failures and were suspended from fatigue testing. Indenters presenting cracks or fractures were replaced.

### Subsurface damage analysis

**Figure 2:**
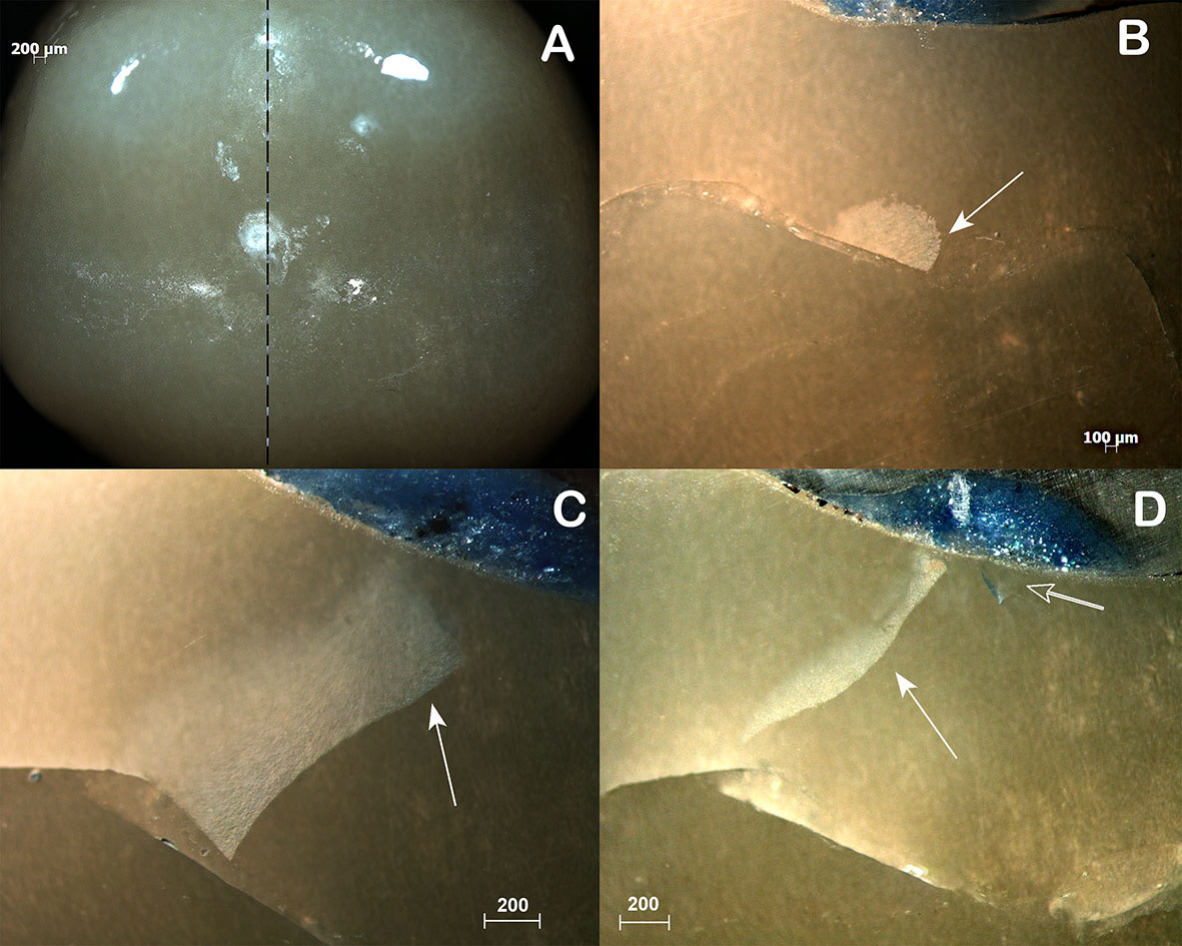
B, C and D depict side views from a LD crowns polished through the entire damage area, as shown in A (occlusal view, 0.8x). In B it is possible to identify a crack that seems to originate from the cementation surface (filled arrow). In C, the crack is propagating further (filled arrow). Finally in D, considering the angle relative to the occlusal surface and the presence of another similar crack (outlined arrow), we concluded it was an inner cone crack extending to the cementation surface (B/C/D Magnification 2x)

The specimens that survived the mechanical test received a layer of gingival barrier (Top Dam, FGM; Joinville, SC, Brazil) on the contact facets, in order to identify the contact area. Later, they were embedded in epoxy resin (Resina Epóxi RD6921, Redelease; São Paulo, SP, Brazil), sectioned with a diamond saw (Extec Corp; Enfield, CT, USA) and serially polished with silicon carbide papers (400, 600, 1200, 2000, 2500 grit) under water cooling. Sectioning started on the mesial surface, far from the contact area, and the crowns were grinded from the mesial to the distal surface with 400 silicon paper polishing and carefully inspected under a stereomicroscopy (MZ6, Leica; Wetzlar, Germany). When any subsurface damage was found the specimen was polished (600, 1200, 2000, 2500 grit to provide better quality images) and photographed under the stereomicroscopy, using a built-in camera (Hitachi CCTV HV-720E, Hitachi; Tokyo, Japan). To ensure subsurface damage was thoroughly analyzed and photographed, the entire indentation area was grinded, polished and photographed to allow for a complete damage inspection (Figure 2). Damage was classified considering the microscope image that shows the cracks in its totality.

**Figure 3:**
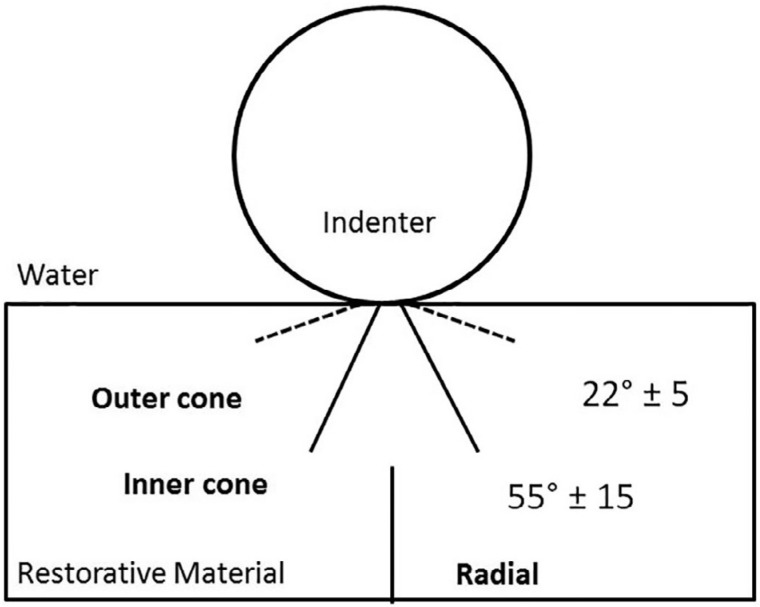
Schematic of contact damage. Outer cone cracks originate around the contact area and typically present an angle of 22±5° relative to free surface. For inner cone cracks, the measure is 55±15°. Radial cracks originate from the cementation surface and propagate sideways and upwards. Viewed from below, they are star-shaped. However, from side view we can it is possible to identify one of its arms propagating towards the contact area

Damage modes were classified into (1) no damage, (2) outer cone cracks, (3) inner cone cracks, (4) inner cone cracks reaching the cementation surface and (5) radial cracks according to damage location and angle relative to the free surface^1^ ^,^ ^2^ ^,^ ^20^ (Figure 3). Scores (0 to 5) were assigned according to subsurface damage severity. Debonded crowns were considered failures and excluded from subsurface damage analysis. Cohesive and catastrophic fractures were scored as failures, as well as radial cracks and inner cone cracks that reached the cementation surface, due to their potential to lead to bulk fracture.

### Statistical analysis

Survival data was subjected to Fisher's exact test (α=0.05). Damage modes were subjected to Mann-Whitney test (α=0.05) using the application software SigmaPlot (Systat Software Inc.; San Jose, CA, USA)

## Results

**Figure 4:**
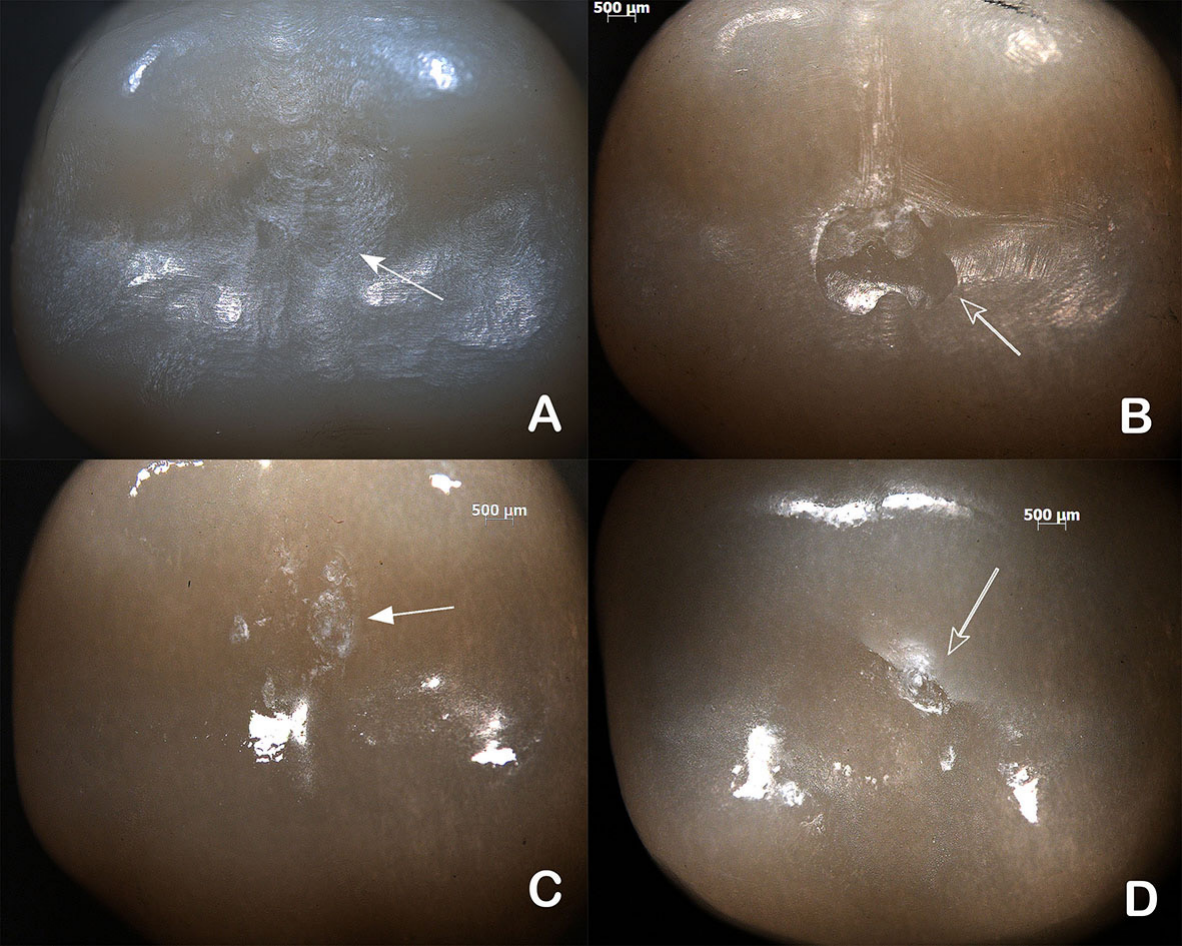
Occlusal view of surface damage after 2 million cycles (0.8x). Small (A) and large (C) wear facets in RNC crowns cycle. Wear facets (C) and crack (D) in LD crowns after 2 million cycles

There were no debonding, catastrophic or cohesive fractures either in the inspections or after the completion of the test. Resin nanoceramic (RNC) specimens showed wear facets of variable sizes (Figures 4a and 4b) and no visible cracks. Lithium disilicate (LD) crowns showed wear facets and removal of glaze (Figure 4c) and two crowns presented cracks in the occlusal surface, detected at the 1 million cycle inspection (Figure 4d).

**Figure 5:**
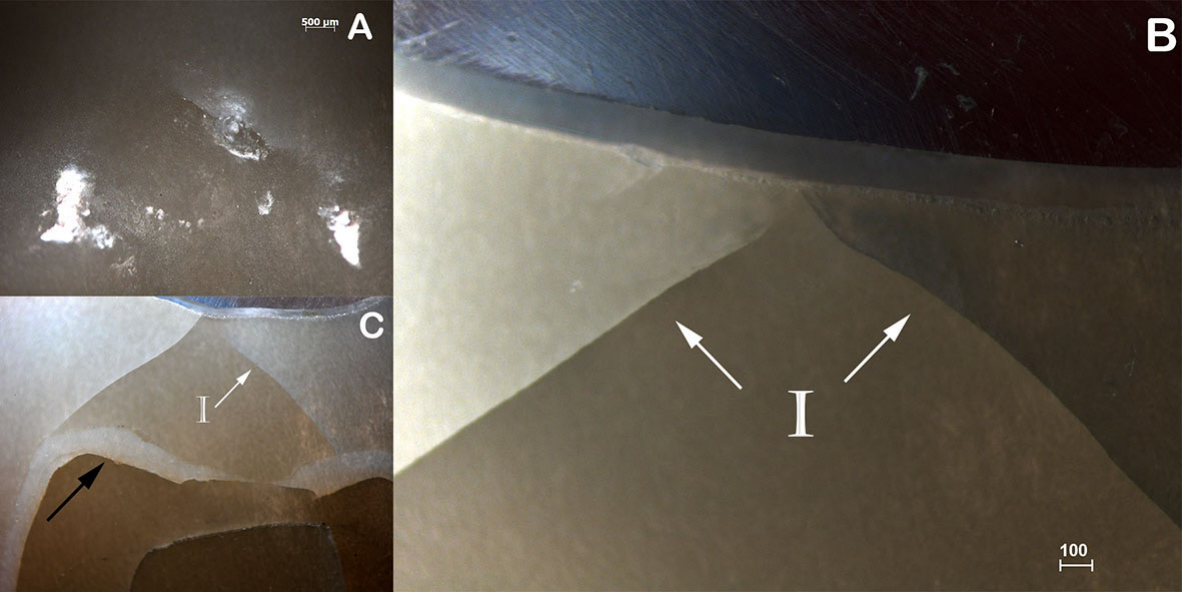
A) Occlusal damage in LD crown (1.25x). B) On the side view of the polished specimen (4x), subsurface damage analysis showed contact-induced inner cone cracks (I). C) Inner cone cracks extending to the cementation surface (I), black arrow shows resin cement layer (1.6x)

**Figure 6:**
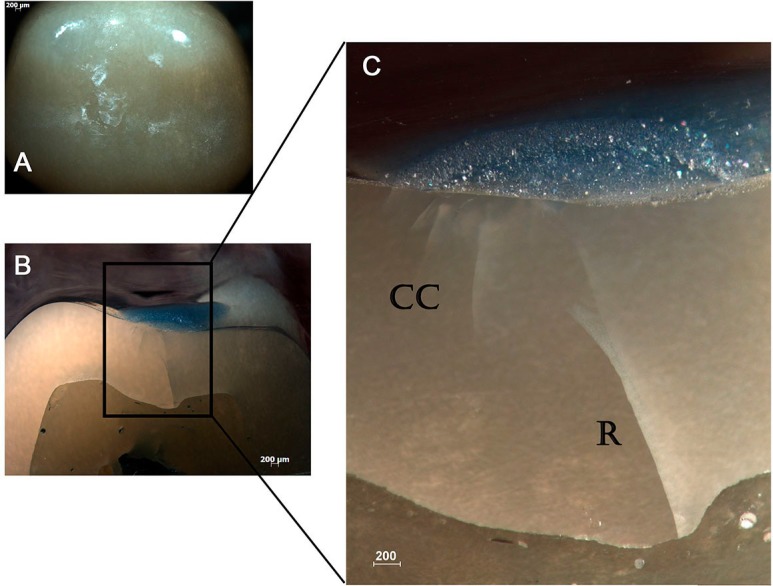
A) Occlusal damage in LD crown (0.8x). B) Side view of LD crown (0.8x). C) Side view (2.5x) showing partial cone (CC) and flexure-induced radial (R) cracks

**Figure 7:**
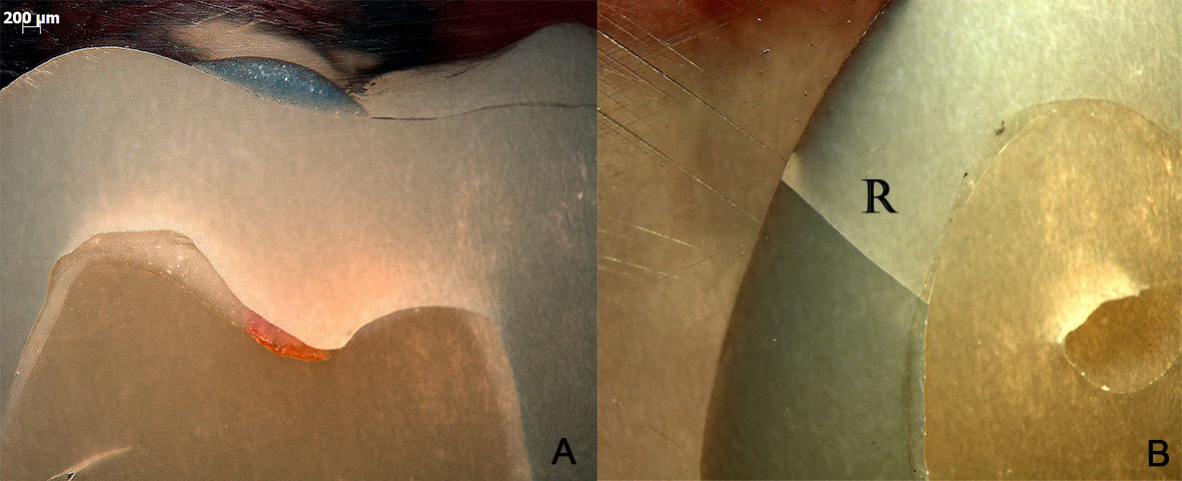
Side view of RNC crowns. In A (0,8x), no damage was detected, however in B (1.6x) a radial crack (R) extends through the entire thickness, in the buccal surface

**Figure 8:**
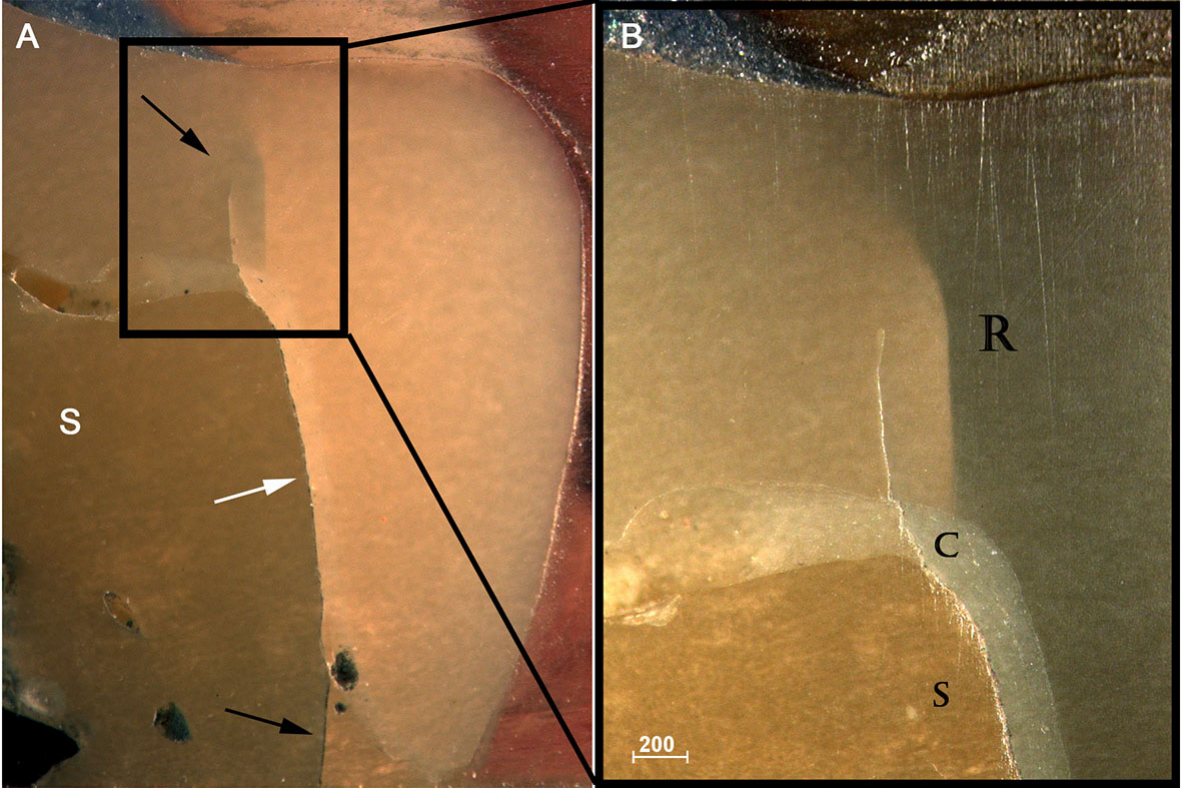
A) Side view (1,6x) of RNC crown showing a radial crack that propagated up- and downwards (black arrows) through the interface (white arrow) of composite substrate (S) and resin cement. B) 2.5x magnification showing the radial crack (R), cement (C) and composite substrate (S)

**Figure 9:**
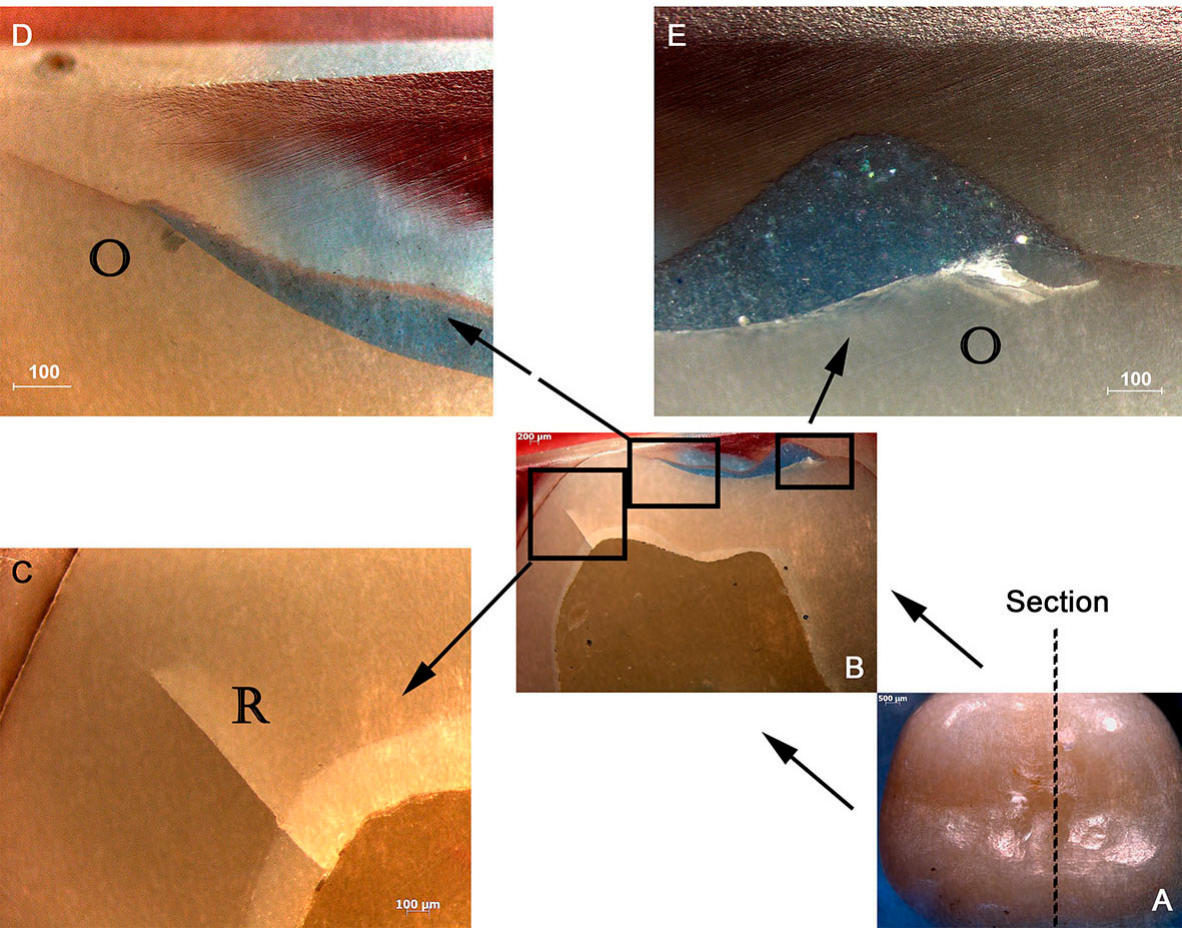
Damage modes in RNC crowns. A) Occlusal view (0.8x) whose section is shown in B. B) Side view that is shown in high magnification. In C (4x) we identify a flexure induced radial crack (R) on buccal face. D and E (4x) show outer cone cracks around indentation area

**Figure 10:**
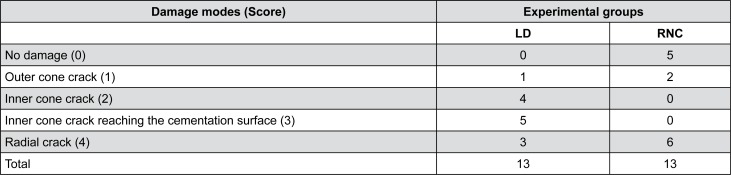
Damage modes by groups

Subsurface damage analysis revealed that inner cone cracks were the dominant crack system mechanism for LD crowns, occurring in 9 crowns. In 5 of them, the inner cone crack reached the cementation surface, which would eventually result in crown fracture (Figure 5). Two crowns presented radial cracks (Figure 6). RNC crowns showed distinct damage modes: 5 crowns presented no detectable damage (Figure 7), however 5 presented radial cracks (Figure 8). Outer and inner cone cracks were present (Figure 9). Damage modes distribution and their respective scores are shown in Figure 10.

Considering subsurface damage analysis, six LD crowns (46.2%) and seven RNC crowns (53.8%) survived, with no statistical difference in fatigue survival (*p*=1.0) or subsurface damage modes between groups (*p*=0.459).

**Figure 11:**
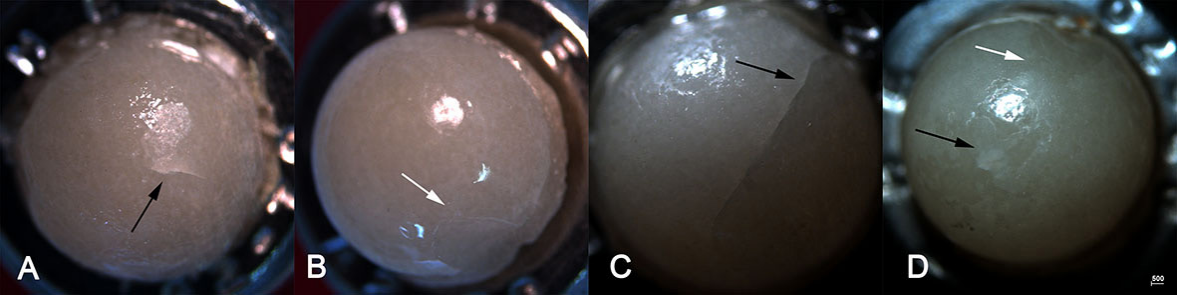
Lithium disilicate indenters (0,8x) A and B loaded LD crowns, C and D loaded RNC crowns. A) Black arrow show small contact crack. B) White arrow show chipping starting in the fixture base. C) Black arrow shows large crack that started in fixture base. D) Black arrow shows removal of glaze, white arrow show chipping starting in the fixture base

In general, lithium disilicate indenters loading RNC crowns presented no damage. Indenters loading LD crowns presented discrete wear facets and removal of the glaze (Figures 11a and 11b). Minor cracks were detected after 500,000 cycles but were followed up during the entire test. One indenter presented a large crack and one presented cohesive fracture; both cracks started close to the fixture base and were not related to contact damage (Figures 11c and 11d).

## Discussion

The null hypothesis that there would be no influence of restorative material on the fatigue survival of lithium disilicate and resin nanoceramic monolithic posterior crowns was confirmed. In this study, LD and RNC crowns presented similar fatigue survival to a 2 million cycles challenge with constant 0-350 N load at 2 Hz; presenting no debonding, cohesive or catastrophic failures. Carvalho, et al.^6^ (2014) investigated the fatigue resistance of 1.5 mm LD and RNC crowns and reported statistically similar failure rates despite applying different fatigue parameters.

Previous studies also reported no fractures of LD crowns after fatigue tests.^21^ ^–^ ^23^ Fatigue failure of LD crowns occurred under high loads and fatigue was not an acceleration factor for failure.^7^ ^,^ ^10^ The results of the current study are in accordance to clinical performance, since bulk fracture and chipping occur in only 3.8% of crowns in 5 years^3^ as a result of a gradual and slow fatigue process. A recent study based in almost 35,000 restorations estimates only 10% of LD single crowns will fail after 20.9 years.^24^ Therefore, it is plausible that fractures do not occur in feasible time under physiological loads *in vitro*.

The promising clinical and mechanical performance of LD crowns can be attributed to lithium disilicate crystals, interlocked needle-like and resistant structures that correspond to 70% volume of this glass ceramic. Crystal arrangement and compressive stresses generated around crystals contribute to crack deflection,^8^ while the reduction of glassy matrix reduces its fatigue susceptibility.^4^ The result is the higher flexural strength and fracture toughness among glass ceramics.^5^ ^,^ ^8^

Apparently, RNC crowns are not affected by damage accumulation.^25^ Shembish, et al.^13^ (2016) subjected teeth supported by 2 mm RNC crowns to fatigue and reported no failures even after 1700 N loads. Clinical performance of Lava Ultimate restorations is unknown, but resin composite is considered unsuitable for crowns in the posterior area, due to its unstable aesthetics, wear and biofilm accumulation.^26^ ^,^ ^27^ Controversially, clinical studies on resin composite crowns report acceptable survival rates varying from 87% to 96%, fracture and wear are mentioned as complications.^26^ ^–^ ^28^

When fatigue tests do not result in failure, the crowns are usually subjected to single load to fracture (SLF). Although this test provides useful data on strength degradation, SLF does not necessarily represent failure in fatigue.^29^ SLF produces fractures under incorrect stress states and high loads, incompatible with masticatory forces.^30^ In turn, subsurface damage analysis can provide information on failure modes of tooth-supported monolithic crowns.

Subsurface damage analysis revealed outer and inner cone cracks, and radial cracks in LD crowns (Figures 5 and 6). Radial cracks are associated to flexure tensile stresses. They start in the cementation surface beneath the contact area, propagating sideways and upwards^31^ and can reach the occlusal/outer surface. Inner cone cracks are induced by contact damage and assisted by water pumping. They appear in wet environments and propagate downwards at higher velocity than outer cone cracks, at a steep angle. They can reach the core ceramics and result in chipping or delamination of ceramic veneer.^1^

Thus, radial cracks have been reported as responsible for bulk fracture in all ceramic crowns,^30^ while chipping or delamination in bilayer crown systems are attributed to inner cone cracks are said to cause failure by.^1^ In this study, however, deep inner cone cracks reached the cementation surface (Figure 5), suggesting competing failure modes may operate and contribute to bulk fracture in monolithic lithium disilicate crowns.^7^

With regard to RNC crowns, previous studies reported distinct failure modes, probably due to different study designs. Carvalho, et al.^6^ (2014) reported catastrophic failure in RNC crowns, probably due to the high loads, once the fractures also involved subjacent dentin. Bonfante, et al.^14^ (2015) reported cohesive fractures in implant-supported RNC crowns, however performed a step-stress fatigue test with an indenter sliding in mesiolingual cusp, an area that clearly provides less support to restorative material. Shembish, et al^13^ (2016) observed partial inner cone cracks and short radial cracks in only 2 from 15 crowns, after a step-stress fatigue test, with sliding contact at the distobuccal cusp. However, they evaluated subsurface damage by sectioning in a single area instead of polishing through the entire specimen.

In the present study, most resin nanoceramic crowns presented contrasting outcomes: while radial cracks occurred in 5 from 13 crowns, other 5 crowns did not show any type of detectable damage. Radial cracks penetrated both the cement layer and supporting composite (Figure 8), or propagated through the entire crown thickness (Figure 7), which would probably lead to bulk fracture. Surprisingly, two of these radial cracks occurred far from the indentation area (Figures 7 and 9), which suggests some lateral movement of the indenter.

With regards to flexural strength, lithium disilicate glass ceramics is capable of withstanding higher stress before failure when compared to resin composite^5^. However, we could speculate that under similar loads in fatigue, other properties ensure comparable performance between RNC and LD. Resin nanoceramic (Lava Ultimate) fatigue strength can be attributed to high filler content, low elastic modulus, good flexural resistance and high Weibull modulus. High filler content improves the fatigue resistance of resin composites, since it decreases the amount of organic matrix and is more susceptible to water sorption, fatigue and strength degradation.^32^ Additionally, the combination of low elastic modulus and good flexural strength (higher than feldspathic and leucite reinforced ceramics) deliver an increased ability to withstand loading by undergoing more elastic deformation before failure. The combination of these properties can be translated into a property known as modulus of resilience. RNC presents higher modulus of resilience than ceramic materials and is consequently capable of absorbing more energy before deforming and/or failing.^33^ In addition, the Weibull modulus (*m*) of Lava Ultimate is higher than e.max CAD.^5^ Although LD may withstand higher loads, non-homogeneously distributed flaws in the ceramic material (that can be microstructural or processing flaws) may act as crack initiators and contribute in decreasing the load to failure. Moreover, when a crack is present, the stress necessary for its propagation is equivalent, since the materials display comparable fracture toughness according to the manufacturers (2 MPa√m for RNC vs. 2 – 2.5 MPa√m for LD). All these factors may compensate for comparable fatigue performance and failure modes that, although different in origin and mechanisms, seem to equally contribute to catastrophic failure of monolithic single crowns.

The present study tested anatomic specimens in water at 37°C under 0-350 N at 2 Hz in order to simulate an oral environment.^1^ ^,^ ^29^ ^–^ ^32^ Such load and frequency conditions were established for being close to masticatory function.^34^ To our knowledge, no previous study subjected LD and RNC crowns to 2 million cycles or more, however, as any *in vitro* experiment, the present study presents limitations. First of all, there is no scientific evidence of correlation between number of cycles in *in vitro* fatigue tests and clinical performance.^35^ Consequently, it is not possible to correlate the survival rates found in this test with clinical survival rates after a certain time. The use of human enamel indenters could represent the clinical situation; however, obtaining theses indenters involves a series of technical and ethical issues. Lithium disilicate indenters were used as an alternative, as they present modulus and wear resistance close to dental enamel.^8^ Additionally, they meet the requirement for using indenters of equal modulus between opposing occlusal contacts when performing contact fatigue tests.^36^

RNC crowns presented fatigue resistance comparable to LD crowns, however, this resin composite is no longer indicated for full crowns due to debonding. Even so, bonding strategies for RNC should be investigated in order to ensure acceptable clinical performance for both partial and full coverage restorations. Future research should also focus on other aspects that could influence the clinical longevity of RNC restorations, such as wear resistance, color stability, surface roughness and biofilm accumulation.

## Conclusions

Monolithic resin nanoceramic and lithium disilicate crowns presented comparable fatigue strength, which suggests RNC crowns can be an alternative treatment for posterior areas.

The materials tested presented different damage modes: resin nanoceramic seems to be more susceptible to flexure-induced radial cracks, while lithium disilicate crowns presented radial and inner cone cracks. Although distinct, both damage modes showed potential to cause failure by bulk fracture in monolithic LD and RNC crowns.

## References

[1] Rekow D, Thompson VP (2007). Engineering long term clinical success of advanced ceramic prostheses. J Mater Sci Mater Med.

[2] Lawn B, Bhowmick S, Bush MT, Qasim T, Rekow ED, Zhang Y (2007). Failure modes in ceramic-based layer structures: a basis for materials design of dental crowns. J Am Ceram Soc.

[3] Sailer I, Makarov NA, Thoma DS, Zwahlen M, Pjetursson BE (2015). All-ceramic or metal-ceramic tooth-supported fixed dental prostheses (FDPs)? A systematic review of the survival and complication rates. Part I: Single crowns (SCs). Dent Mater.

[4] Della Bona A, Mecholsky JJ, Anusavice KJ (2004). Fracture behavior of lithia disilicate- and leucite-based ceramics. Dent Mater.

[5] Belli R, Geinzer E, Muschweck A, Petschelt A, Lohbauer U (2014). Mechanical fatigue degradation of ceramics versus resin composites for dental restorations. Dent Mater.

[6] Carvalho AO, Bruzi G, Giannini M, Magne P (2014). Fatigue resistance of CAD/CAM complete crowns with a simplified cementation process. J Prosthet Dent.

[7] Silva NR, Bonfante EA, Martins LM, Valverde GB, Thompson VP, Ferencz JL (2012). Reliability of reduced-thickness and thinly veneered lithium disilicate crowns. J Dent Res.

[8] Guess PC, Schultheis S, Bonfante EA, Coelho PG, Ferencz JL, Silva NR (2011). All-ceramic systems: laboratory and clinical performance. Dent Clin North Am.

[9] Coelho PG, Silva NR, Bonfante EA, Guess PC, Rekow ED, Thompson VP (2009). Fatigue testing of two porcelain-zirconia all-ceramic crown systems. Dent Mater.

[10] Guess PC, Zavanelli RA, Silva N, Bonfante EA, Coelho PG, Thompson VP (2010). Monolithic CAD/CAM lithium disilicate versus veneered Y-TZP crowns: comparison of failure modes and reliability after fatigue. Int J Prosthodont.

[11] Gracis SE, Nicholls JI, Chalupnik JD, Yuodelis RA (1990). Shock-absorbing behavior of five restorative materials used on implants. Int J Prosthodont.

[12] Harada A, Nakamura K, Kanno T, Inagaki R, Örtengren U, Niwano Y (2015). Fracture resistance of computer-aided design/computer-aided manufacturing-generated composite resin-based molar crowns. Eur J Oral Sci.

[13] Shembish FA, Tong H, Kaizer M, Janal MN, Thompson VP, Opdam NJ (2016). Fatigue resistance of CAD/CAM resin composite molar crowns. Dent Mater.

[14] Bonfante EA, Suzuki M, Lorenzoni FC, Sena LA, Hirata R, Bonfante G (2015). Probability of survival of implant-supported metal ceramic and CAD/CAM resin nanoceramic crowns. Dent Mater.

[15] Schepke U, Meijer HJ, Vermeulen KM, Raghoebar GM, Cune MS (2016). Clinical bonding of resin nano ceramic restorations to zirconia abutments: a case series within a randomized clinical trial. Clin Implant Dent Relat Res.

[16] Schepke U, Lohbauer U, Meijer HJ, Cune MS (2018). Adhesive failure of lava ultimate and lithium disilicate crowns bonded to zirconia abutments: a prospective within-patient comparison. Int J Prosthodont.

[17] Peumans M, Valjakova EB, De Munck J, Mishevska CB, Van Meerbeek B (2016). Bonding effectiveness of luting composites to different CAD/CAM materials. J Adhes Dent.

[18] Ab-Ghani Z, Jaafar W, Foo SF, Ariffin Z, Mohamad D (2015). Shear bond strength of computer-aided design and computer-aided manufacturing feldspathic and nano resin ceramics blocks cemented with three different generations of resin cement. J Conserv Dent.

[19] Ferracane JL (2013). Resin-based composite performance: are there some things we can't predict?. Dent Mater.

[20] Kim JW, Kim JH, Thompson VP, Zhang Y (2007). Sliding contact fatigue damage in layered ceramic structures. J Dent Res.

[21] Seydler B, Rues S, Müller D, Schmitter M (2014). *In vitro* fracture load of monolithic lithium disilicate ceramic molar crowns with different wall thicknesses. Clin Oral Investig.

[22] Zhao K, Wei YR, Pan Y, Zhang XP, Swain MV, Guess PC (2014). Influence of veneer and cyclic loading on failure behavior of lithium disilicate glass-ceramic molar crowns. Dent Mater.

[23] Heintze SD, Cavalleri A, Zellweger G, Büchler A, Zappini G (2008). Fracture frequency of all-ceramic crowns during dynamic loading in a chewing simulator using different loading and luting protocols. Dent Mater.

[24] Belli R, Petschelt A, Hofner B, Hajtó J, Scherrer SS, Lohbauer U (2016). Fracture rates and lifetime estimations of CAD/CAM all-ceramic restorations. J Dent Res.

[25] Bonfante EA, Almeida EO, Lorenzoni FC, Coelho PG (2015). Effects of implant diameter and prosthesis retention system on the reliability of single crowns. Int J Oral Maxillofac Implants.

[26] Vanoorbeek S, Vandamme K, Lijnen I, Naert I (2009). Computer-aided designed/computer-assisted manufactured composite resin versus ceramic single-tooth restorations: a 3-year clinical study. Int J Prosthodont.

[27] Ohlmann B, Bermejo JL, Rammelsberg P, Schmitter M, Zenthöfer A, Stober T (2014). Comparison of incidence of complications and aesthetic performance for posterior metal-free polymer crowns and metal-ceramic crowns: results from a randomized clinical trial. J Dent.

[28] Jongsma LA, Kleverlaan CJ, Feilzer AJ (2012). Clinical success and survival of indirect resin composite crowns: results of a 3-year prospective study. Dent Mater.

[29] Rekow ED, Silva NR, Coelho PG, Zhang Y, Guess P, Thompson VP (2011). Performance of dental ceramics: challenges for improvements. J Dent Res.

[30] Kelly JR (1999). Clinically relevant approach to failure testing of all-ceramic restorations. J Prosthet Dent.

[31] Zhang Y, Sailer I, Lawn BR (2013). Fatigue of dental ceramics. J Dent.

[32] Lohbauer U, Belli R, Ferracane JL (2013). Factors involved in mechanical fatigue degradation of dental resin composites. J Dent Res.

[33] Awada A, Nathanson D (2015). Mechanical properties of resin-ceramic CAD/CAM restorative materials. J Prosthet Dent.

[34] Palinkas M, Nassar MSP, Cecílio FA, Siéssere S, Semprini M, Machado-de-Sousa JP (2010). Age and gender influence on maximal bite force and masticatory muscles thickness. Arch Oral Biol.

[35] Nawafleh N, Hatamleh M, Elshiyab S, Mack F (2016). Lithium disilicate restorations fatigue testing parameters: a systematic review. J Prosthodont.

[36] Bhowmick S, Mélendez-Martínez JJ, Hermann I, Zhang Y, Lawn BR (2007). Role of indenter material and size in veneer failure of brittle layer structures. J Biomed Mater Res B Appl Biomater.

